# Cochlear gene therapy with ancestral AAV in adult mice: complete transduction of inner hair cells without cochlear dysfunction

**DOI:** 10.1038/srep45524

**Published:** 2017-04-03

**Authors:** Jun Suzuki, Ken Hashimoto, Ru Xiao, Luk H. Vandenberghe, M. Charles Liberman

**Affiliations:** 1 Department of Otology and Laryngology, Harvard Medical School, Boston, MA 02115, USA; 2 Eaton-Peabody Laboratories, Massachusetts Eye & Ear Infirmary, Boston, MA 02114, USA; 3 Department of Otorhinolaryngology-Head and Neck Surgery, Tohoku University Graduate School of Medicine, Sendai, Miyagi 980-8574, Japan; 4 Grousbeck Gene Therapy Center, Schepens Eye Research Institute and Massachusetts Eye & Ear Infirmary, Boston, MA 02114, USA; 5 Ocular Genomics Institute, Department of Ophthalmology, Harvard Medical School, Boston, MA 02114, USA; 6 Harvard Stem Cell Institute, Harvard University, Cambridge, MA 02138, USA

## Abstract

The use of viral vectors for inner ear gene therapy is receiving increased attention for treatment of genetic hearing disorders. Most animal studies to date have injected viral suspensions into neonatal ears, via the round window membrane. Achieving transduction of hair cells, or sensory neurons, throughout the cochlea has proven difficult, and no studies have been able to efficiently transduce sensory cells in adult ears while maintaining normal cochlear function. Here, we show, for the first time, successful transduction of all inner hair cells and the majority of outer hair cells in an adult cochlea via virus injection into the posterior semicircular canal. We used a “designer” AAV, AAV2/Anc80L65, in which the main capsid proteins approximate the ancestral sequence state of AAV1, 2, 8, and 9. Our injections also transduced ~10% of spiral ganglion cells and a much larger fraction of their satellite cells. In the vestibular sensory epithelia, the virus transduced large numbers of hair cells and virtually all the supporting cells, along with close to half of the vestibular ganglion cells. We conclude that this viral vector and this delivery route hold great promise for gene therapy applications in both cochlear and vestibular sense organs.

In recent years, the use of viral vectors for gene therapy in the inner ear has received increasing attention as a way to treat either acquired or hereditary sensorineural hearing loss (for review see refs 1, 2, 3, 4, 5). The sensory epithelia of the inner ear are promising sites for viral therapies because they are isolated, in part, by a blood-labyrinth barrier, and because they are bathed in a continuous series of fluid-filled spaces encased in bone. Over the past decade, viral vectors have been used in animal studies to enhance survival of cochlear neurons after hair cell destruction by gene transfer with neurotrophic factors^6^,^7^,^8^,^9^,^10^,^11^. Viruses have also been used to deliver wildtype copies of mutant genes in mouse models of genetic disorders of synaptic transmission^5^, mechanoelectric transduction^12^, stereocilia assembly^13^, or stria vascularis function^14^. Several of these studies have shown functional rescue of some fraction of sensory cells in these otherwise profoundly deaf animal models.

Many of the recent attempts at virally mediated gene transfer in the inner ear have used adeno-associated viruses (AAV) as vectors. AAVs are attractive candidates, because they are not linked with disease, they are minimally immunogenic, and they can achieve efficient and long-lived transgene expression^15^. In the inner ear, many naturally occurring AAV variants appear to preferentially target hair cells, which also makes them attractive vectors^5^,^13^,^16^,^17^,^18^,^19^,^20^,^21^. However, to date, no AAV serotype has been shown to target hair cells throughout the entire cochlear spiral in adults.

In addition to the need for vectors for cellular transduction, one of the challenges for inner ear gene therapies is achieving local delivery without damaging the delicate structures of the inner ear. In the adult, the sensory epithelia of the cochlear and vestibular systems are encased in the hardest bone in the body, and, as highly sensitive mechanosensors, can be easily damaged by mechanical manipulations such as drilling or fluid injections. The three commonly used routes for drug/virus delivery into the inner ear are 1) via the round window membrane, at the base of the cochlear spiral, where it faces the middle ear space, 2) via a hole drilled through the cochlear capsule into the cochlear fluid spaces near the round window, or 3) through a hole drilled into the posterior semicircular canal where it runs near the external surface of the skull^16^,^17^,^18^,^20^,^22^,^23^,^24^,^25^. In our experience in animal models, injection through the semicircular canals is the least traumatic to the cochlea. To date however, these approaches have not yielded widespread hair cell transduction in the adult ear without elevating cochlear thresholds.

Here we show, in a mouse model, that AAV injections into the posterior semicircular canal via an optimized procedure in the adult mouse can be made without causing any thresholds shift, suprathreshold dysfunction, or hair cell damage, even to the delicate high-frequency regions at the extreme basal tip of the cochlear spiral. The procedure requires only a post-auricular incision followed blunt dissection of the thin muscle layers overlying the temporal bone, revealing clear surface landmarks indicating the position of the canal. A small hole in the skull is made with a 26 gauge hypodermic, and virus is delivered into the canal through a microcatheter. In order to maximize transduction efficiencies, a “designer” AAV vector, AAV2/Anc80L65, was tested based on promising results in organotypic explant cultures. AAV2/Anc80L65 is an AAV of which the main capsid proteins were synthesized to approximate the ancestral sequence state of AAV1, 2, 8, 9 and several other serotypes^15^,^26^. Our studies show that AAV2/Anc80L65 via posterior semicircular canal injections targets virtually 100% of IHCs throughout the cochlear spiral, as well as a significant fraction of OHCs. In the vestibular system, our injections appear to transduce all of the supporting cells in all the sensory epithelia of the saccule, utricle and semicircular canals. This combination of technical approaches should be useful for future development of gene therapies for sensorineural hearing loss.

## Results

### Injections into normal cochleas

**Figure 1:**
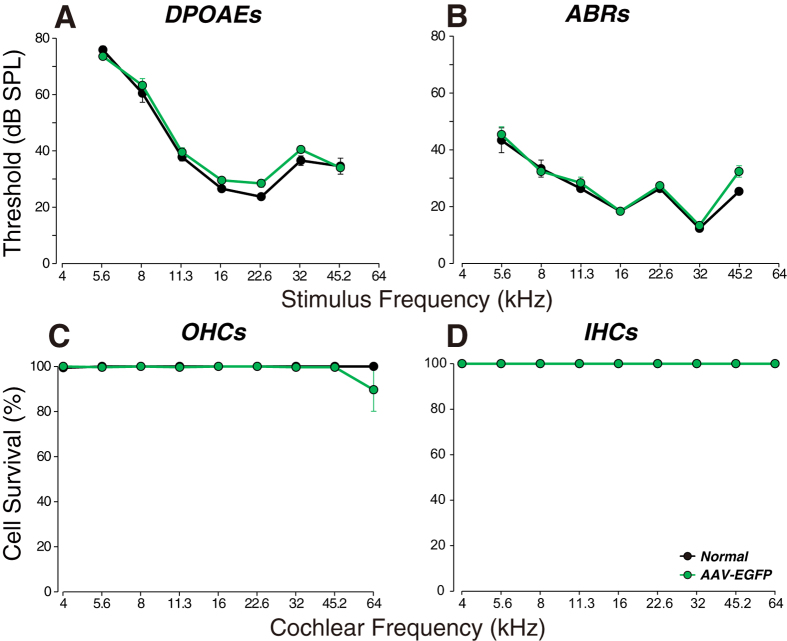
Virus injection via the posterior semicircular canal in the adult mouse does not cause permanent threshold shifts or cochlear damage. (**A**,**B**) Cochlear thresholds, measured by DPOAEs or ABRs (group means ± SEMs) 2 wks after injection. (**C**,**D**) Outer and inner hair cell loss (group means ± SEMs) for each group, as seen immediately after the cochlear function measures in (**A**,**B**). Group sizes for (**A**,**B**) were: Normal = 5; AAV-EGFP = 5. Group sizes for (**C**,**D**) were the same except Normal = 3. Symbol key in (**D**) applies to all panels.

There are two minimally invasive tests of cochlear function that give a sensitive readout of the functional state of the cochlear sensory epithelium: distortion product otoacoustic emissions (DPOAEs) and auditory brainstem responses (ABRs). As seen in Fig. 1A,B, injection of AAV2/Anc80L65 into the posterior semicircular canals caused no significant changes in DPOAE or ABR thresholds at any of the test frequencies, as measured 2 wks post injection (P = 0.21 and 0.27, respectively, by two-way ANOVA). The lack of threshold shifts in either of these measures suggests that there was no significant damage to mechanoelectric transduction in sensory cells anywhere along the cochlear spiral. Indeed, subsequent histopathological analysis showed no significant inner hair cell (IHC) loss anywhere, and no outer hair cell (OHC) loss except for a modest loss (~10%) at the extreme basal tip of the cochlear spiral, in regions tuned to frequencies higher than 45.2 kHz (our highest test frequency). This OHC damage was only seen in one of 5 cases.

**Figure 2:**
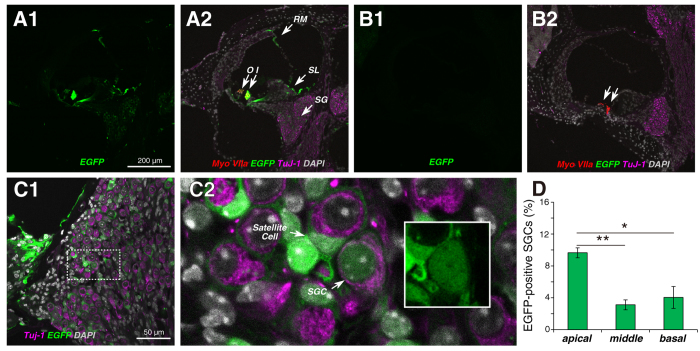
In the cochlea, the strongest transduction is seen in IHCs. (**A1**,**A2**) Two views of the same section through the middle of the cochlea show EGFP signals in IHCs (I) and OHCs (O), interdental cells and fibrocytes in the spiral limbus (SL), cells of Reissner’s membrane (RM) and cells in the spiral ganglion (SG). (**B1**,**B2**) Two views of the same section show no EGFP signal in an uninjected ear. Positions of IHCs and OHCs shown by arrows. (**C1**,**C2**) EGFP signal is seen throughout the spiral ganglion (**C1**), which at higher power (**C2**) is seen to be in both satellite cells and spiral ganglion cells (SGCs). Inset to C2 (white box) shows the labeled SGC without the TuJ-1 channel to show EGFP signal in nucleus and cell body. (**D**) Percent of transduced SGCs in three cochlear regions (means ± SEMs from two sections from each of three ears). Scale bar in (**A1**) applies to all (**A**,**B**) panels. Statistical significance is shown by asterisks: * P < 0.05; ** P < 0.01.

To investigate which cochlear cells were transduced by AAV2/Anc80L65, we cut frozen sections of the inner ear and looked for EGFP expression. As shown Fig. 2A1, the brightest cells, by far, were the IHCs, but weaker EGFP signal was also seen in OHCs, in epithelial cells of Reissner’s membrane, in the interdental cells and fibrocytes of the spiral limbus and in Rosenthal’s canal, where cell bodies of cochlear sensory neurons, the spiral ganglion cells, are located. Higher power views of the ganglion region (Fig. 2C1) reveal EGFP signal both in the satellite cells surrounding the spiral ganglion cells, as well as in the nuclei and cell body of the spiral ganglion cells themselves (Fig. 2C2), which are immunostained using β-tubulin (TuJ-1), a general neuronal marker. Most of the label was in the satellite cells: counts in apex middle and basal half turns of the cochlea (Fig. 2D) showed that fewer than 10% of the spiral ganglion cells were EGFP-positive. There was no EGFP signal detectable in uninjected cochleas (Fig. 2B1).

**Figure 3:**
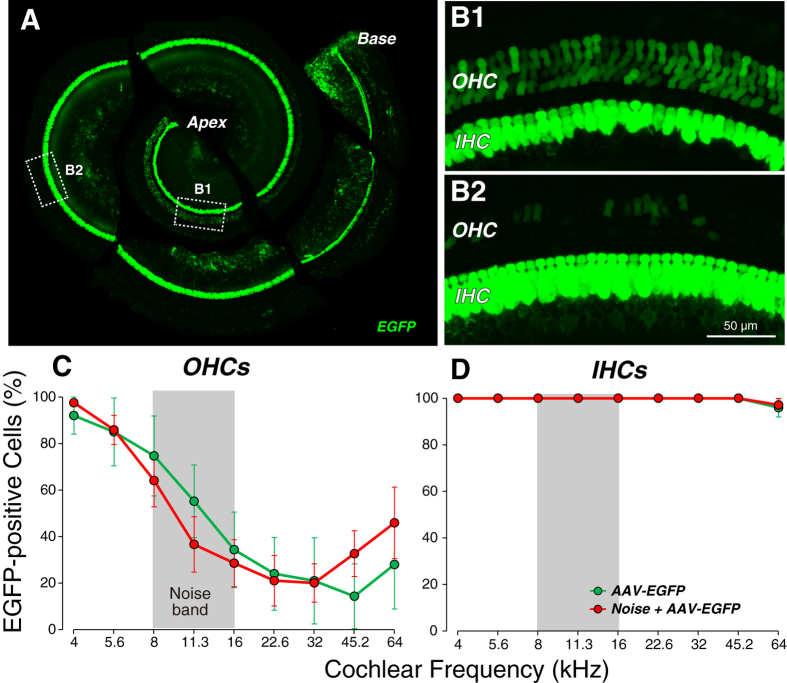
Transduction efficiency is essentially 100% in IHCs throughout the cochlear spiral. (**A**) Low-magnification view of an injected cochlea, dissected into six pieces, showing EGFP signal from the apical to the basal tip. Dashed boxes show regions selected for high-magnification views. B1,B2: High-magnification views of the organ of Corti from the boxed regions shown in (**A**). Scale bar in B2 also applies to B1. All imaged in this montage were acquired with the same settings. (**C**,**D**) Percentage of transduced outer (**C**) and inner (**D**) hair cells as a function of cochlear place/frequency (group means ± SEMs). Group sizes were: AAV-EGFP = 5; Noise + AAV-EGFP = 8. Symbol key in (**D**) also applies to (**C**).

To more quantitatively evaluate the transduction efficiency of AAV2/Anc80L65 in hair cells, we analyzed the cochlear sensory epithelium as microdissected whole mounts. The photomontage in Fig. 3A clearly shows EGFP expression all along the cochlear spiral from base to apex. Higher power views of the sensory epithelium (Fig. 3B1) show that IHCs express much higher levels of EGFP than OHCs. In the micrographs shown, every IHC is EGFP-positive. We counted the percentage of EGFP-positive IHCs and OHCs at nine equally spaced positions along the cochlear spiral in 5 injected cochleas: as shown in Fig. 3D, literally every IHC was transduced except for a very few at the basal tip, whereas the OHC targeting tended to decrease from apex to base.

**Figure 4:**
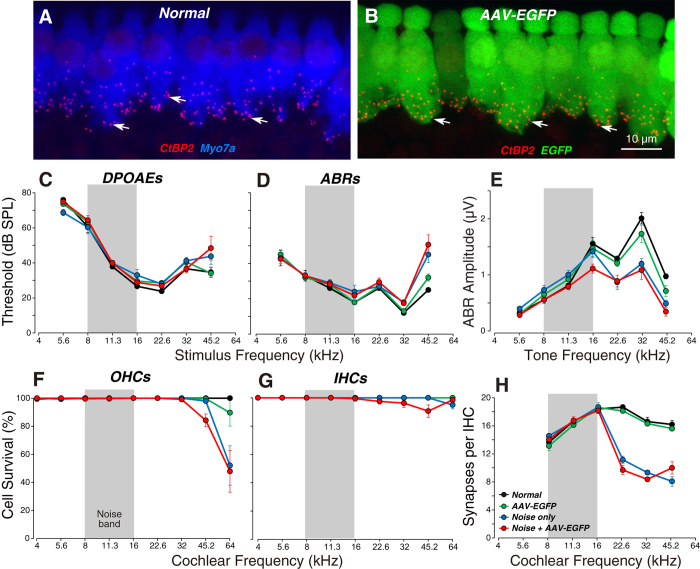
Viral transduction does not reduce synaptic counts in the IHC area or the suprathreshold amplitude of ABR wave 1, in either normal or noise-stressed animals. (**A**,**B**) Maximal projections from the 22.6 kHz region immunostained for CtBP2 (red) to reveal the synapses between auditory nerve terminals and IHCs. Scale bar in (**B**) applies to (**A**). (**C**,**D**) Mean DPOAE and ABR thresholds (±SEMs) for all four experimental groups. (**E**) Mean ABR wave 1 amplitudes (±SEMs) for suprathreshold levels (60–80 dB SPL). (**F**,**G**) Mean counts of OHC and IHC survival for all four experimental groups. (**H**) Mean IHC synaptic counts (±SEMs) for all four experimental groups. Group sizes were: Normal = 5; AAV-EGFP = 5; Noise only = 5, and Noise + AAV-EGFP, n = 8 in (**C–E**): Normal = 3; AAV-EGFP = 5; Noise only = 5, and Noise + AAV-EGFP, n = 8 in (**F**,**G**): Normal = 3; AAV-EGFP = 5; Noise only = 5, and Noise + AAV-EGFP, n = 7 in (**H**). Threshold data and hair cells counts for Normal and AAV-EGFP groups are reproduced from Figure 1 for comparison to the noise-stressed groups.

Since the IHCs seemed to be the major target of the virus, we sought to further probe the functional state of the transduced cells. OHCs function as biological motors, amplifying sound-evoked cochlear vibrations. Thus, DPOAE and ABR thresholds are sensitive measures of OHC function. IHCs, on the other hand, provide the synaptic drive to the primary sensory fibers of the cochlear nerve. ABR and DPOAE thresholds are quite insensitive to silencing of IHCs or their synapsing cochlear neurons, so long as that loss does not exceed ~80%^27^. The most sensitive measure of IHC and neuronal function is the suprathreshold amplitude of the first wave of the ABR, which represents the summed activity of cochlear nerve fibers^28^. As shown in Fig. 4E, viral transduction caused no significant decrement in these suprathreshold amplitudes, at any of the test frequencies spanning the cochlear spiral (P = 0.89 by two-way ANOVA). Correspondingly, there was also no change in the number of synaptic contacts (Fig. 4H; P = 0.79 by two-way ANOVA), as assessed by counting presynaptic ribbons immunostained for CtBP2, a major constituent of these ribbon synapses, and post-synaptic glutamate-receptor patches^29^,^30^.

### Injections into damaged cochleas

Translation of viral gene therapies to sensorineural hearing loss in humans will require the ability to target of cells within a damaged inner ear. One potential application has been suggested by recent animal work showing that one of the major debilitating effects of noise exposure is retraction of the peripheral axons of cochlear nerve fibers^28^,^30^ and that driving transgenic overexpression of neurotrophins in hair cells or supporting cells can partially rescue of the noise-induced neuropathy^31^. Anticipating future gene therapy experiments with neurotrophin-expressing vectors, we wondered if viral transduction of noise-stressed cochleas would be as safe and effective as in the normal cochlea.

We noise-stressed two groups of animals by exposing them to a mid-frequency noise band for 2 hrs at intensities near 100 dB sound pressure level (SPL). Prior studies have shown that this exposure causes a dramatic acute elevation of cochlear threshold^28^,^30^,^32^. However, thresholds recover in a few days, and there are only minor permanent threshold shifts, and OHC loss, and these losses are seen only at the highest test frequencies (Fig. 4C,D,F). Nevertheless, this noise stress leads to a permanent retraction of ~50% of IHC synapses throughout the basal half of the cochlea (Fig. 4H).

Here we show that injection of viral particles into these noise-stressed ears via the posterior canal route causes no additional DPOAE and ABR threshold shifts (Fig. 4C,D; P = 0.98 and 0.97, respectively, by two-way ANOVA), no additional loss of IHCs or OHCs (Fig. 4F,G; P = 0.88 and 0.66, respectively, by two-way ANOVA), no additional decrement in ABR Wave 1 amplitudes (Fig. 4E; P = 0.72 by two-way ANOVA from 16–45.2 kHz) and no additional loss of IHC synapses (Fig. 4H; P = 0.95 by two-way ANOVA from 16–45.2 kHz). Furthermore, prior noise stress to the sensory epithelium had no significant effect on the transduction efficiency in either OHCs or IHCs (Fig. 3C,D; P = 0.85 and 0.81, respectively, by two-way ANOVA).

### Transduction in the vestibular epithelia

**Figure 5:**
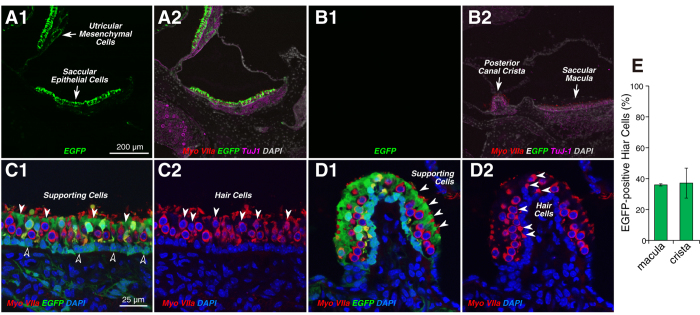
In the vestibular epithelia, virus transduction is seen in both maculae and cristae, and in both hair cells and supporting cells. **A1**,**A2**: EGFP signal was seen in both saccular and utricular sensory epithelia, and, less strongly, in underlying mesenchymal cells. (**B**) No EGFP-expressing cells were observed in the vestibular epithelia in a control case. **C1**,**C2** A higher power view of the utricular macula, with (**C1**) and without (**C2**) the EGFP channel, show that virtually all (myosin VIIa-negative) supporting cells are transduced, whereas only a subset of the hair cells are transduced. White-fill arrowheads in (**C1** and **C2**) point to some transduced supporting cells and hair cells, respectively. Black-fill arrowheads point to the nuclei of some EGFP-positive supporting cells. (**D1**,**D2**) A higher power view of the crista ampularis, with (**D1**) and without (**D2**) the EGFP channel, show that virtually all (myosin VIIa-negative) supporting cells are transduced, whereas only a subset of the hair cells are transduced. (**E**) Percent of transduced vestibular hair cells in the macula or the crista: mean (±SEM) from two sections from each of three ears. Scale bars in (**A1** and **C1**) apply to all panels in their respective rows.

Although our major focus is on applications to sensorineural hearing loss, we were also interested in qualitatively assessing the targeting efficiency of the AAV2/Anc80L65 virus in the vestibular system. As shown Fig. 5A1,A2, 2 wks after virus injection, there is widespread EGFP expression throughout the sensory epithelia of the utricle and saccule. As in the cochlea, there was never any EGFP signal in the vestibular epithelia of uninjected controls (Fig. 5B1). In higher power views of both the utricular macula (Fig. 5C1) and the crista ampullaris (Fig. 5D1), it is clear that virtually all supporting cells are transduced, whereas only a subset of the hair cells (~35%) is EGFP positive (Fig. 5E). In the utricular macula, the supporting cell nuclei form a continuous row at the mesenchymal side of the epithelium (dark-fill arrows in Fig. 5C1), while the hair cells form two roughly staggered rows closer to the endolymphatic surface of the organ. There was a clear predilection for transduction of type II hair cells, which generally constitute the cells with nuclei closer to the endolymph side of the epithelium^33^.

**Figure 6:**
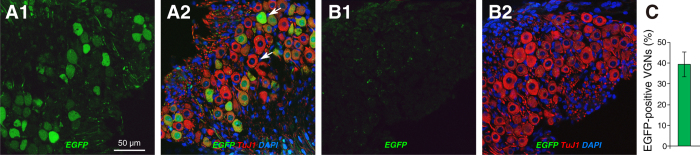
In the vestibular ganglion, many (TuJ-1-positive) ganglion cells are transduced, whereas the transduction of satellite cells is not clear. (**A1**,**A2**,**B1**,**B2**) Confocal micrographs of sections through the vestibular ganglion in a virus-injected case (**A1**,**A2**) and an uninjected control (**B1**,**B2**). Each image is shown as a 3-channel merge (**A2**,**B2**) and as the EGFP channel only (**A1**,**B1**). (**C**) Percent of transduced ganglion cells: mean (±SEM) from two sections from each of three ears. Scale bar in (**A1**) applies to all panels.

The cell bodies of vestibular nerve fibers are found in Scarpa’s Ganglion in the internal auditory meatus. As shown in Fig. 6A1, virus injection led to widespread transduction of vestibular ganglion cells (39.3%; Fig. 6C) and no obvious transduction of satellite cells. This pattern differs markedly from the spiral ganglion, where <10% of the neurons were transduced and many satellite cells were EGFP positive (Fig. 2C,D).

By 2 wks after virus injection, we observed no circling behavior or other gait abnormalities in the mice that might indicate gross vestibular dysfunction. To provide a more quantitative measure of vestibular function, we also measured vestibular evoked potentials (VsEPs)^34^: mean VsEP thresholds in virus-injected ears (−12.5 dB re 1 g/ms; n = 3) were within 1.5 dB of that seen in control ears (−11.0 dB re 1 g/ms; n = 5).

## Discussion

### Adeno-associated virus and cochlear targeting efficiencies

AAV is one of the most frequently used viral vectors in gene therapy, because it can efficiently infect a variety of post-mitotic tissues and result in stable gene expression, with minimal vector-related immune response and toxicity^2^,^20^,^23^. Due to these advantages, AAV vectors have been safely used in the clinical studies over the past decades, which more recently led to demonstrable efficacy for monogenic disease approaches targeting muscle and retina^35^,^36^,^37^.

In the inner ear, a number of animal studies, using several AAV serotypes, have injected virus into the neonatal cochlea, primarily aiming to rescue hearing in mouse models of autosomal recessive forms of sensorineural hearing loss by gene augmentation^5^,^12^,^13^,^14^. All these studies have found that AAV transduces IHCs more than any other cochlear cell type, however, the transduction efficiencies varied. One study of neonatal AAV1 injections reported transduction of 100% of IHCs^5^. Another neonatal study using AAV2/1 cites 60% transduction among IHCs, when averaged across the entire cochlea^12^, with lower transduction rates in the cochlear apex (~50%) than the base (~85%). In contrast, a neonatal study using AAV8 reports transduction of only 15% of IHCs^13^. Transduction efficiency also declines with postnatal age: one study reports ~100% of cochlear sections showed at least one transfected IHC transduction when AAV1 injections were made near postnatal day 0, while only ~40% were positive when AAV1 injections were made at postnatal day 10 with the same amount of virus^5^. Two studies have provided proof of principle for cochlear gene therapy by documenting some functional rescue in the adult ears of these otherwise profoundly deaf mutant lines^5^,^12^. All these neonatal studies have injected virus through the round window, but none has been able to assess resultant cochlear damage, because none of these experimental subjects begin with functioning cochlear sensory epithelia.

Others have injected GFP-expressing AAV vectors into the normal adult ear, as in the present study, to evaluate the effectiveness of different delivery routes or viral serotypes in either mice^16^,^17^,^20^,^24^, rat^25^ or guinea pig^38^. Although the most successful of these prior studies reports near 100% transfection of IHCs in the base of the cochlea after round window injection^20^, most report significantly less than that, e.g. ~30%^17^ and ~80%^38^, and all report only 0–20% transduction in the cochlear apex^17^,^20^,^38^. Similarly, although four prior studies have looked at virus delivery via a posterior canal route^22^,^23^,^24^,^25^, none convincingly demonstrated a high targeting efficiency for hair cells throughout the cochlear spiral.

In this study, we evaluated a unique AAV capsid, called Anc80L65, designed to mimic an ancestral form of several AAV serotypes in order to minimize antigenic similarity to naturally circulating AAV that poses a problem in clinical translation of AAV gene therapies^26^. Anc80L65 was pursued as it demonstrated compelling transduction characteristics in organotypic explant cultures. By injecting viral vector through the posterior semicircular canal, we were able to achieve IHC transduction rate of essentially 100% throughout the entire cochlea from base to apex (Fig. 3D), and high OHC transduction rates in the apical turn (Fig. 3C). Considering that two of our injected ears showed very high OHC transduction rate in all cochlear regions (Supplemental Figure S1), we suspect that we can increase the OHC transduction by adjusting vector suspension volume, viral titer or injection speed without jeopardizing cochlear function. We examined reporter gene expression 2 wks after AAV2/Anc80L65 injection into the inner ear. In prior study of AAV2/Anc80L65 injection into liver, muscle or retina, continued expression has been documented for 4 wks post-injection^26^. Further investigation is needed to evaluate the longer-term stability of these viral transgenes.

Even in the IHC area, the EGFP signal appeared stronger in the apical than in the basal tip (Fig. 3). This observation suggests that viral entry into the hair cells may be predominately through the basilar membrane from the scala tympani side rather than through Reissner’s membrane from the scala vestibuli side. An injection into perilymph of the posterior canal should produce higher concentrations, for scala vestibuli, in the base than the apex, but higher concentrations for scala tympani in the apex than the base. From the posterior canal, the fluid will first enter the cochlea via scala vestibuli in the basal turn, which is separated from the hair cell compartment by Reissner’s membrane. As further fluid is injected, the suspension will spiral up the cochlea to enter scala tympani in the apex after traveling through the helicotrema. Once in scala tympani, virus particles can access the basolateral surfaces of the hair cells after penetrating the basilar membrane, which tracer studies have shown does not present a significant barrier, even to relatively large molecules^39^.

Our posterior canal injections were highly effective in targeting supporting cells in all the vestibular sensory epithelia, and in transducing the type II hair cells more so than type I hair cells. This is perhaps unexpected, since type II hair cells are more similar to OHCs, at least with respect to their innervation patterns^40^.

### Cochlear gene delivery without jeopardizing inner ear function

The inner ear is located deep within the petrous portion of the temporal bone, so the delivery of drugs or viruses is more challenging than, for example, in the eye. Several delivery strategies have been developed that access the cochlear fluids directly from that portion of the cochlear basal turn facing the middle-ear space. These approaches include: (1) a cochleostomy into either scala tympani or scala media^17^,^18^,^22^,^41^,^42^, (2) injection through the round window membrane^5^,^17^,^20^,^43^, (3) or injection onto the surface of the round window, with or without manipulations to increase penetration^38^,^44^,^45^,^46^. The approach to the posterior canal differs in that the access is not through the middle ear^22^,^23^,^24^,^25^.

Although approaches through the middle ear may be practical in human, where the relevant structures are relatively large, the middle and inner ear spaces in mouse are so small, and the inner ear so delicate, that the surgical access to, and/or injection into, the cochlear fluids seems to unavoidably cause cochlear damage^2^,^16^,^17^,^22^, unless injections are made prior to postnatal day 5^42^. Although Kilpatrick *et al*. reported a cochleostomy approach with controlled release of AAVs into scala media of adult mice, they could not avoid cochlear injury at high frequency regions (≥32 kHz)^18^. In the trans-round window approach, the viral vector can be delivered to scala tympani without drilling the cochlea^5^,^17^,^20^,^43^. However, this approach in adult mice also causes significant ABR threshold shifts^17^. Although Liu *et al*. reported no ABR threshold shifts after round window injections in adult mice, they did not measure responses at frequencies >24 kHz, which leaves the basal third of the spiral untested^20^. Injections onto, rather than through, the round window may be the least traumatic of the middle-ear approaches to virus delivery^2^. Although collagenase or hyaluronic acid pretreatments have been reported to promote efficient viral penetration through the round window, without introduction of permanent threshold shifts^38^,^44^,^45^,^46^, to date, such an approach has only been successful into the inner ear of the guinea pig and neonatal mice.

In this study, we injected via the posterior semicircular canal in adult mice. This approach does not require opening the middle ear, and prior studies have reported minimal threshold shifts^22^,^23^,^24^,^25^,^47^. However, prior studies have reported that transduced cells mainly localize in the vestibular system^22^,^23^,^24^, and the canalostomy approach has not previously been used for cochlear gene therapy. Prior studies have also noted disadvantages of the canalostomy approach including: (1) the difficulty of distinguishing whether the cannula is in (inner/membranous) endolymph or (outer/bony) perilymph spaces, and (2) leakage of the inner ear fluid and vector suspension between the bone and the cannula^22^. In this study, we cannot be certain about the precise position of the cannula tip. However, its diameter nearly equals that of the bony canal (Supplemental Figure S2D), thus it is unlikely that injections were ever restricted to the endolymph compartment. Any uncertainty as to the position of the cannula caused no apparent problems, and we saw no leakage around the cannula using cyanoacrylate adhesives instead of carboxylate cement.

We showed that virus injection via the canalostomy approach can target supporting cells and type II hair cells in all the vestibular epithelia as well as IHCs and OHCs throughout the cochlear spiral, without any obvious vestibular dysfunction or changes in vestibular evoked potentials, and without any cochlear threshold shifts or hair cell damage, even in the basalmost cochlear regions. Moreover, detailed physiological and histological analyses revealed that suprathreshold amplitudes of ABR wave 1 and IHC synapse numbers are also unchanged. Using this sensitive measure of IHC function, we conclude that is possible to target essentially every IHC along the cochlear spiral without any measurable dysfunction. Such capability may prove useful in eliciting re-innervation of de-afferented hair cells by viral transduction of IHCs with genes for neurotrophins such as NT-3^31^. Thus, the canalostomy approach to delivery of a resurrected ancestral AAV should be generally useful for preclinical studies of gene therapy for sensorineural hearing loss in adult mice. Because the semicircular canal can be also accessed in humans, analogous approaches may also be a promising method for future cochlear gene therapy in the hearing impaired.

## Methods

### Vector constructs and production

AAV2/Anc80L65 vector constructs were produced to carry an AAV2 ITR flanked genome encoding CMV driven EGFP, a Woodchuck Hepatatis Virus Regulatory Element (WPRE) and a bovine Growth Hormone poly-adenylation site. Vectors were produced as previously described^26^,^48^ by transfection of 3 constructs (dF6 adenoviral helper, pAAV2/Anc80L65 encoding AAV2 rep and Anc80L65 cap genes, and the ITR flanked transgene plasmid) in HEK293 cells. After 3 days, cells and media were harvested^49^, filtered and purified using iodixanol sedimentation on an ultracentrifuge. Final vector preparations were buffered in dPBS for injection (3.84 × 10^12^ genome copy/mL).

### Animals and experimental design

Male CBA/CaJ mice were obtained from Jackson Laboratories at 6 wks of age. Animals were randomly assigned to one of four experimental groups. All animals underwent cochlear function testing at 9 wks, immediately followed by cochlear fixation and histopathological analysis. An AAV-EGFP group was injected with virus at 7 wks; a Noise-only group was exposed to a noise designed to destroy cochlear synapses but not hair cells^30^,^32^ at 7 wks; a Noise + AAV-EGFP group was exposed to noise at 7 wks and injected with virus 24 hrs later; and a Normal group received neither noise nor virus injection. All experimental procedures were approved by the Institutional Animal Care and Use Committee of the Massachusetts Eye and Ear Infirmary and conducted in accordance with the NIH Guide for the Care and Use of Laboratory Animals.

### Noise exposure

Mice were exposed, awake and unrestrained, to octave-band noise (8–16 kHz) for 2 hrs at 98 dB sound pressure level (SPL) in a reverberant sound-exposure box at 7 wks^30^,^32^. Mice were held within acoustically transparent wire cages on a rotating platform. The noise waveform was generated digitally using a fifth-order Butterworth filter, amplified through a power amplifier (Crown D75A), and delivered by a loudspeaker (JBL2446H) coupled to an exponential horn in the roof of the box. Sound levels were confirmed in the center of the cage with a ¼” Bruel and Kjaer condenser microphone before each exposure and varied by less than 1 dB in the cage space.

### Cochlear function testing

ABRs and DPOAEs were recorded as described previously^32^. The animals were anesthetized with an intraperitoneal injection of ketamine (100 mg/kg) and xylazine (20 mg/kg) and placed in an acoustically and electrically shielded room maintained at 32 °C. Acoustic stimuli were delivered through a custom acoustic system consisting of two miniature dynamic earphones used as sound sources (CDMG15008-03A, CUI) and an electret condenser microphone (FG-23329-PO7, Knowles) coupled to a probe tube to measure sound pressure near the eardrum. Custom LabVIEW software controlling National Instruments 24-bit soundcards (6052E) generated all stimuli and recorded all responses. For ABRs, stimuli were 5 ms tone pips (0.5 ms cos^2^ rise-fall) at frequencies from 5.6 to 45.2 kHz (in half-octave steps) delivered in alternating polarity at 35/s. Electrical responses were collected via needle electrodes at the vertex and at the ventral edge of the pinna with a ground reference near the tail, amplified 10,000X with a 0.3–3 kHz passband, and averaged with 1024 responses at each SPL. Responses were collected for stimulus levels in 5-dB steps from 10 dB below threshold up to 80 dB SPL. ABR threshold was defined as the lowest sound level at which a reproducible waveform could be observed. Wave 1 amplitude was defined as the difference between the maximum of the wave 1 peak and the minimum of the subsequent trough. For DPOAEs, the cubic distortion product 2*f*_1_ − *f*_2_ was measured in response to primaries f_1_ and f_2_ (frequency ratio *f*_2_/*f*_1_ = 1.2, and level ratio L1 = L2 + 10), where f_2_ varied from 5.66 to 45.25 kHz in half-octave steps. Primaries were swept in 5 dB steps from 10 to 80 dB SPL (for *f*_2_). The DPOAE at 2*f*_1_ − *f*_2_ was extracted from the ear canal sound pressure after both waveform and spectral averaging. DPOAE threshold was computed by interpolation as the primary level (*f*_1_) required to produce a DPOAE of 0 dB SPL.

### Vestibular function testing

For VsEPs, electrical responses were collected under the same anesthetic regimen, and with the same electrode configuration, amplifier and passbands, as for ABR testing. To stimulate the vestibular sense organs, the mouse’s head was tightly coupled to a mechanical shaker (Labworks, Inc. Model ET132-203), which was driven by a power amplifier (Crown D75A), to accelerate the ear along the dorso-ventral axis. The electrical signal driving the shaker was a 16 Hz sawtooth (produced digitally via the same National Instruments soundcards used for the ABR measurements) with a slowly rising phase and rapid 2 ms return to baseline. The system was calibrated via an accelerometer securely coupled to the drive rod of the shaker. By convention, 0 dB was defined as the stimulus producing a “jerk” of 1 g/ms^34^. As for ABRs, electrical responses to 1024 stimuli of alternating polarity were averaged at each stimulus level. Stimuli were varied in intensity from −20 dB to +5 dB *re* 1 g/ms. Threshold was defined by visual inspection of stacked waveforms.

### Nanoinjection system

A 30 mm polyethylene tube (PE10, ID 0.011 inches, OD 0.024 inches, Becton Dickinson) was fused to a 20 mm polyimide tube (ID 0.0039 inches, OD 0.0049 inches, Microlumen) using cyanoacrylate glue. The other end of the polyethylene tube was connected to a glass micropipette (#4878, World Precision Instruments) using cyanoacrylate glue. After the glue dried, we filled the tubes and the micropipette with mineral oil, and connected the micropipette to the Nanoliter 2010 injector controlled by MICRO4 Controller (World Precision Instruments).

### Surgical approach

Mice were anesthetized with ketamine (100 mg/kg) and xylazine (20 mg/kg). The fur behind the left ear was shaved with a razor and sterilized with 10% povidone iodine. A15-mm postauricular skin incision was made. After exposing the facial nerve and the sternocleidomastoid muscle by blunt dissection, a part of muscle was cut proximally. The muscles covering the temporal bone were separated and retracted dorsally using 4-0 vicryl sutures (Supplemental Figure S2A), exposing the bony wall of the posterior semicircular canal, whose margin is clearly visible as a dark stripe in the bone (Supplemental Figure S2B). A small hole was made near the canal with a 26-gauge hypodermic needle (Supplemental Figure S2B). We waited 5 minutes for leakage of perilymph to abate, then inserted the tip of the polyimide tube into the canal toward the ampulla (Supplemental Figure S2C). The aperture between the polyimide tube and the hole was sealed with muscle fragments and cyanoacrylate glue (3 M Vetbond Tissue Adhesive), and the tightness of sealing was visually assessed by the lack of fluid leakage. Then, 250 nL of viral suspension was injected at 100 nL/min for 2.5 min; the tube was left in the canal for 5 min after injection. After removing the tube, the hole was plugged with small pieces of muscle, and covered with cyanoacrylate glue. The skin was closed with 5-0 nylon sutures. Total surgical time ranged from 40 to 50 min (Supplementary video S1).

### Cochlear processing and immunohistochemistry for whole mounts

After transcardial perfusion with 4% paraformaldehyde, cochleas were perfused through the cochlear scalae, dissected from the temporal bones, and post-fixed for 2 hrs at room temperature. Cochleas were then decalcified in 0.12 M EDTA at room temperature for 2 days and microdissected into 6 pieces for whole-mount cochlear processing. For immunostaining, cochlear pieces were blocked with 5% normal horse serum in phosphate-buffered saline (PBS) and 0.3% Triton X-100 for 1 hr at room temperature followed by overnight incubation at 37 °C with following primary antibodies diluted in 1% normal horse serum with 0.3% TX: 1) mouse (IgG1) anti-CtBP2 (C-terminal Binding Protein) at 1:200 (#612044, BD Transduction Labs) for quantifying pre-synaptic ribbons; 2) mouse (IgG2a) anti-GluA2 (Glutamate receptor subunit A2) at 1:2000 (#MAB397, Millipore) for quantifying post-synaptic receptor patches; and 3) rabbit anti-Myosin VIIa at 1:200 (#25-6790 Proteus Biosciences) for delineating hair cells. Cochlear pieces were washed and then incubated twice for 60-min at 37 °C in species-appropriate secondary antibodies: 1) Pacific Blue-conjugated goat anti-rabbit IgG at 1:200 (#P10994, Life Technologies); 2) Alexa Fluor 568-conjugated goat anti-mouse (IgG1) at 1:1000 (#A21124, Life Technologies); 3) Alexa Fluor 647-conjugated goat anti-mouse (IgG2a) at 1:200 (#A21241, Life Technologies). Finally, cochlear pieces were slide mounted using Vectashield (Vector Labs) and coverslipped. Cochlear pieces were imaged at low magnification (×4 objective) using a fluorescent microscope (E800, Nikon), and a custom ImageJ plug-in http://www.masseyeandear.org/research/otolaryngology/investigators/laboratories/eaton-peabody-laboratories/epl-histology-resources/) was used to create a cochlear frequency map.

### Synapse and hair cell counts

Immunostained cochlear whole mounts were imaged at 6 log-spaced cochlear frequency regions via confocal microscopy (TCS SP5, Leica) with a glycerol-immersion 63X objective (1.3 N.A.) and 3.2X digital zoom. For each image stack, the z dimension was sampled at 0.25 μm, with the span adjusted to include all synaptic elements in the xy field of view. Each z-stack included 8~12 adjacent IHCs, and two contiguous z-stacks were obtained in each frequency location in each ear. To identify and count hair cell synapses, image stacks were ported to image processing software (Amira, Visage Imaging). Pre-synaptic ribbons in each z-stack were isolated and counted using the “*connected components”* function in Amira. IHCs in each stack were counted using staining from the anti-Myosin VIIa. To quantitatively assess the juxtaposition of pre-synaptic ribbons and post-synaptic receptor patches, we used custom software that extracts the voxel space within 1 μm around each ribbon and produces a thumbnail array of these miniature projections, that can be scanned to count synapses versus orphan ribbons. For IHC and OHC counts, we acquired z-stacks at the same 7 log-spaced cochlear frequency locations, with the same glycerol-immersion 63X objective, but without digital zoom, such that each stack contained 20~30 IHCs and 60~90 OHCs across the three rows. IHC and OHC survival and vector transduction were assessed using the anti-Myosin VIIa immunostain and EGFP signal.

### Immunohistochemistry for frozen sections

After overnight treatment in a 30% sucrose in PBS at 4 °C, decalcified cochleas were embedded in OCT (Tissue-Tek) and were frozen. Sections in 10 μm thickness were cut parallel to the modiolus on a cryostat (CM3050S, Leica Instruments), mounted on glass slides and stored at −20 °C. For immunohistochemistry, tissue sections were rinsed in PBS and were blocked with 5% normal horse serum in PBS and 0.3% Triton X-100 for 1 hr at room temperature followed by overnight incubation at 4 °C with following primary antibodies diluted in 1% normal horse serum with 0.3% TX: 1) mouse anti-Tuj-1 (Neuron-specific class III beta-tubulin) at 1:500 (MMS-435P, Covance) for neurons; 2) rabbit anti-Myosin VIIa at 1:250 (#25-6790 Proteus Biosciences) for hair cells. Tissue sections were washed and then incubated for 90-min at room temperature in species-appropriate secondary antibodies: 1) Alexa Fluor 568-conjugated donkey anti-rabbit (IgG) at 1:500 (#A10042, Life Technologies); 2) Alexa Fluor 647-conjugated donkey anti-mouse (IgG) at 1:250 (#A31571, Life Technologies). Finally, cochlear pieces were slide mounted using Vectashield Mounting Medium with DAPI (H-1200, Vector Labs) and coverslipped. Immunofluorescence images were obtained using a laser-scanning confocal microscope (TCS SP5, Leica). For calculating the percentage of EGFP-positive spiral ganglion cells, we acquired two spiral ganglion images of apical, middle, and basal turns of each case (n = 3) with the glycerol-immersion 63X objective. For calculating the percentage of EGFP-positive vestibular ganglion cells, we acquired two vestibular ganglion images from each case (n = 3). For calculating the percentage of EGFP-positive vestibular hair cells, we acquired two images of the macula and the crista of each case (n = 3).

### Statistical analysis

Statistical analyses were conducted using Kaleidagraph (Synergy Software) for one-way analysis of variance (ANOVA), followed by a post hoc Tukey HSD test or Matlab (MathWorks) for two-way ANOVA, followed by a post hoc Tukey HSD test. All of the data are presented as means ± standard errors of the mean (SEMs).

## Additional Information

**How to cite this article:** Suzuki, J. *et al*. Cochlear gene therapy with ancestral AAV in adult mice: complete transduction of inner hair cells without cochlear dysfunction. *Sci. Rep.* **7**, 45524; doi: 10.1038/srep45524 (2017).

**Publisher's note:** Springer Nature remains neutral with regard to jurisdictional claims in published maps and institutional affiliations.

## Supplementary Material

Supplementary Information

Supplementary video S1
