# Corrigendum: Cochlear gene therapy with ancestral AAV in adult mice: complete transduction of inner hair cells without cochlear dysfunction

**DOI:** 10.1038/srep46827

**Published:** 2017-05-22

**Authors:** Jun Suzuki, Ken Hashimoto, Ru Xiao, Luk H. Vandenberghe, M. Charles Liberman

Scientific Reports 7: Article number: 4552410.1038/srep45524; published online: 04 03 2017; updated: 05 22 2017

The authors forgot to cite previous studies relating to *in vivo* studies in newborn mice. These additional references are listed below as references 1 and 2, and should appear in the text as below.

In the Introduction section,

“In order to maximize transduction efficiencies, a “designer” AAV vector, AAV2/Anc80L65, was tested based on promising results in organotypic explant cultures”.

should read:

“In order to maximize transduction efficiencies, a “designer” AAV vector, AAV2/Anc80L65, was tested based on promising results in organotypic explant cultures and *in vivo* studies in newborn mice^1^,^2^”.
