# Supplementary material for: Cochlear gene therapy with ancestral AAV in adult mice: complete transduction of inner hair cells without cochlear dysfunction
Source: Sci Rep. 2017 Apr 3;7:45524. doi: 10.1038/srep45524 (PMC5377419; doi:10.1038/srep45524)
Supplement: Supplementary Information [file srep45524-s1.pdf]

Supplementary Information

Cochlear gene therapy with ancestral AAV in adult mice: complete transduction of inner hair cells without cochlear dysfunction

Jun Suzuki[1,2,3], Ken Hashimoto[1,2,3], Ru Xiao[4,5], Luk H. Vandenberghe[4,5,6] and M. Charles Liberman[1,2]

[1]Department of Otology and Laryngology, Harvard Medical School, Boston, MA 02115, USA

[2] Eaton-Peabody Laboratories, Massachusetts Eye & Ear Infirmary, Boston, MA 02114, USA

[3] Department of Otorhinolaryngology-Head and Neck Surgery, Tohoku University Graduate School of Medicine, Sendai, Miyagi 980-8574, Japan

[4] Grousbeck Gene Therapy Center, Schepens Eye Research Institute and Massachusetts Eye & Ear Infirmary, Boston, MA 02114, USA

[5] Ocular Genomics Institute, Department of Ophthalmology, Harvard Medical School, Boston, MA 02114, USA

[6] Harvard Stem Cell Institute, Harvard University, Cambridge, MA 02138, USA

Supplementary Figure Legends

Supplemental Figure 1: Transfection efficiency in OHCs can be close to 100%. The percentage of EGFP positive OHCs as a function of cochlear frequency/location in each of the 13 individual cases studied.

Supplemental Figure 2: The surgical approach to the posterior semicircular canal. **A**: View of the left side of the skull after separating and scraping the overlying muscle and fascia. Dashed box is shown at higher magnification in the next panel. **B**: Final view of canalostomy, showing its position relative to the lateral and posterior canals, which are visible through the exposed skull. **C**: Insertion of a polyimide tube into the posterior canal. **D**: Horizontal, plastic section through an un-operated mouse of the same strain and age, showing the posterior canal where it enters the ampulla. The polyimide tube (red; schematized) is drawn to scale: O.D. = 124 μm.

Supplementary video S1: Surgery video (avi file, 51.6 MB)

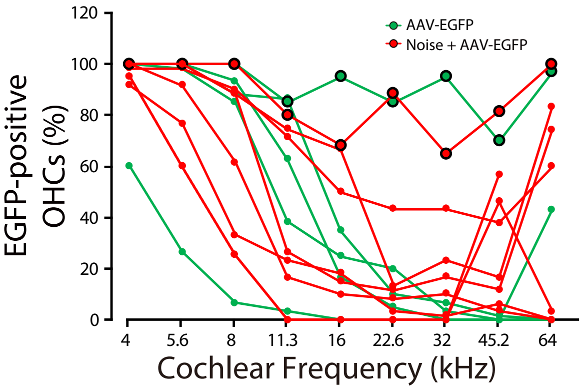

AAV-EGFP
Noise + AAV-EGFP
EGFP-positive OHCs (%)
120
100
80
60
40
20
0
4
5.6
8
11.3
16
22.6
32
45.2
64
Cochlear Frequency (kHz)

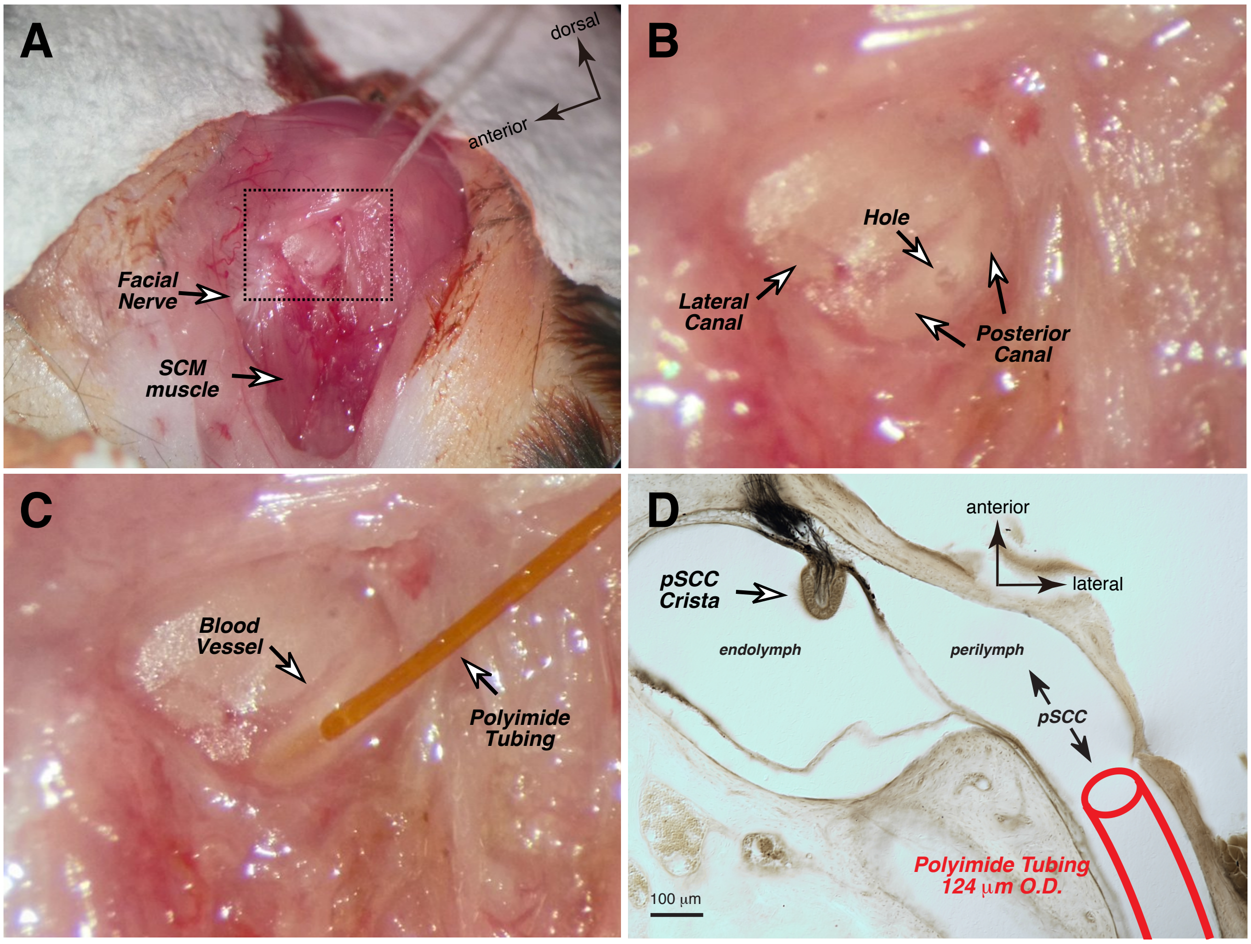
A
dorsal
anterior
Facial Nerve
SCM muscle
B
Hole
Lateral Canal
Posterior Canal
C
Blood Vessel
Polyimide Tubing
D
anterior
lateral
pSCC Crista
endolymph
perilymph
pSCC
Polyimide Tubing
124 μm O.D.
100 μm
